# Regulation of cytochrome P450 2e1 expression by ethanol: role of oxidative stress-mediated pkc/jnk/sp1 pathway

**DOI:** 10.1038/cddis.2013.78

**Published:** 2013-03-21

**Authors:** M Jin, A Ande, A Kumar, S Kumar

**Affiliations:** 1 Division of Pharmacology and Toxicology, School of Pharmacy, University of Missouri-Kansas City, Kansas City, MO, USA

**Keywords:** ethanol, CYP2E1, oxidative stress, PKC/JNK/SP1, astrocytes, monocytes

## Abstract

CYP2E1 metabolizes ethanol leading to production of reactive oxygen species (ROS) and acetaldehyde, which are known to cause not only liver damage but also toxicity to other organs. However, the signaling pathways involved in CYP2E1 regulation by ethanol are not clear, especially in extra-hepatic cells. This study was designed to examine the role of CYP2E1 in ethanol-mediated oxidative stress and cytotoxicity, as well as signaling pathways by which ethanol regulates CYP2E1 in extra-hepatic cells. In this study, we used astrocytic and monocytic cell lines, because they are important cells in central nervous system . Our results showed that 100 mM ethanol significantly induced oxidative stress, apoptosis, and cell death at 24 h in the SVGA astrocytic cell line, which was rescued by a CYP2E1 selective inhibitor, diallyl sulfide (DAS), CYP2E1 siRNA, and antioxidants (vitamins C and E). Further, we showed that DAS and vitamin C abrogated ethanol-mediated (50 mℳ) induction of CYP2E1 at 6 h, as well as production of ROS at 2 h, suggesting the role of oxidative stress in ethanol-mediated induction of CYP2E1. We then investigated the role of the protein kinase C/c-Jun N-terminal kinase/specificity protein1 (PKC/JNK/SP1) pathway in oxidative stress-mediated CYP2E1 induction. Our results showed that staurosporine, a non-specific inhibitor of PKC, as well as specific PKC*ζ* inhibitor and PKC*ζ* siRNA, abolished ethanol-induced CYP2E1 expression. In addition, inhibitors of JNK (SP600125) and SP1 (mithramycin A) completely abrogated induction of CYP2E1 by ethanol in SVGA astrocytes. Subsequently, we showed that CYP2E1 is also responsible for ethanol-mediated oxidative stress and apoptotic cell death in U937 monocytic cell lines. Finally, our results showed that PKC/JNK/SP1 pathway is also involved in regulation of CYP2E1 in U937 cells. This study has clinical implications with respect to alcohol-associated neuroinflammatory toxicity among alcohol users.

Cytochrome P450 (CYP) enzymes comprise a superfamily of heme proteins, which have a major role in phase I metabolic clearance of numerous xenobiotics in the liver.^1^ To a lesser extent, they are also involved in xenobiotic metabolism in other organs, such as intestine, brain, and kidney. CYP2E1 is known to metabolize ethanol in the liver, especially in chronic alcohol users;^2^ however, the persistent use of alcohol and resulting alcohol metabolism is known to cause liver toxicity.^3^ The alcohol metabolism-mediated liver toxicity occurs through the formation of reactive oxygen species (ROS) and the reactive metabolite, acetaldehyde, which ultimately cause DNA damage and lipid and protein oxidations.^4^

Low levels of alcohol in occasional or social mild-to-moderate drinkers are metabolized mainly by alcohol dehydrogenase (ADH), which also appears to cause oxidative stress-mediated liver toxicity.^5^ However, alcohol-inducible CYP2E1 also has an important role in alcohol metabolism and mediate liver impairment among variety of alcohol drinkers.^2^ Although the role of CYP2E1 in alcohol-mediated liver toxicity is well known, similar studies are limited in extra-hepatic cells, especially cells from the CNS. Results from previous studies have led to the suggestion that in neurons and monocytes/macrophages the involvement of CYP2E1 in alcohol metabolism is greater than that of ADH, because ADH is present at very low levels in these cells.^6, 7^ This hypothesis is further strengthened by the fact that ADH is induced far less than CYP2E1 by alcohol in the liver as well as in other organs.^8^ The induction of CYP2E1 by alcohol appears to be through translational, post-translational (protein stabilization), and transcriptional mechanisms.^9^ At low concentrations of alcohol, CYP2E1 exhibits increasing activity and increased protein stability. However, at high concentrations of alcohol, both mRNA and protein expression levels of CYP2E1 are induced. Although post-translational stabilization of CYP2E1 protein and increased activity by alcohol has been described,^9^ the mechanism by which the expression of CYP2E1 is regulated at the level of transcription is poorly understood.

In the brain, CYP2E1 is the only enzyme involved in the non-catalase oxidation of ethanol and ROS production.^10^ Its induction leads to increased lipid peroxidation and apoptosis, resulting in increased permeability of the blood–brain barrier and neurodegeneration.^11^ However, limited information is available on the role of CYP2E1 in ethanol-mediated effects on human astrocytes, which is the predominant cell type in the brain and its major role is to protect neuronal integrity.^12, 13^ Activated astrocytes, especially through increased oxidative stress by alcohol, may cause neuronal damage. Similarly, limited information is available on monocytes with regard to alcohol/CYP2E1. Monocytes infiltrate into the brain and differentiate into microglia and perivascular macrophages, which are also the major cell types in the brain.^14^ This study has been designed to examine the role of CYP2E1 in ethanol-mediated effects on astrocytes and monocytes. Therefore, in this study, we used human SVGA astrocytic and U937 monocytic cell lines to investigate the role of CYP2E1 in ethanol-mediated oxidative stress, apoptosis, cell death, and the mechanism by which ethanol regulates CYP2E1 expression.

## Results

### Role of CYP2E1 in oxidative stress-mediated apoptosis and cell death by ethanol in SVGA astrocytes

**Figure 1:**
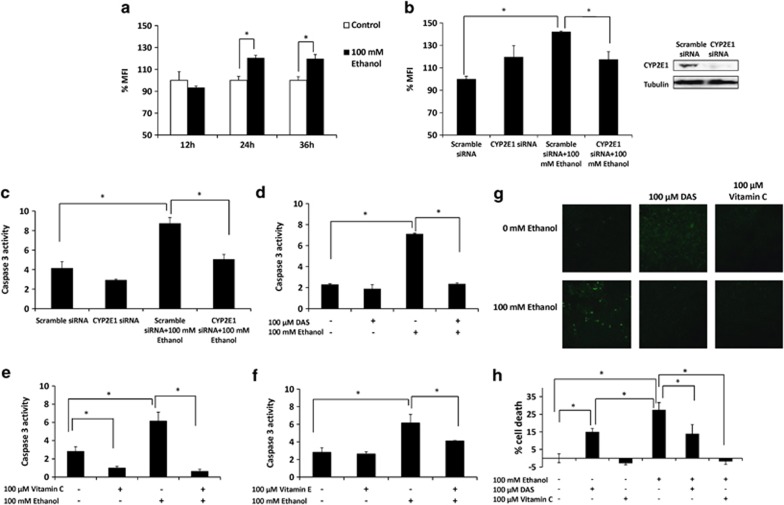
Role of CYP2E1 in oxidative stress-mediated apoptosis and cell death by ethanol in SVGA astrocytes. (**a**) Effect of 100 mℳ ethanol on ROS production in 12–36 h. (**b**) Effect of CYP2E1 small interfering RNA (siRNA) on ethanol-mediated ROS production at 24 h. (**c**) Effect of CYP2E1 siRNA on caspase-3 cleavage activity at 24 h. (**d**) Effect of 100 *μ*M DAS on caspase-3 cleavage activity at 24 h. (**e**) Effect of vitamin C on caspase-3 cleavage activity at 24 h. (**f**) Effect of vitamin E on caspase-3 cleavage activity at 24 h. (**g**) Effect of DAS and vitamin C on DNA fragmentation using TUNEL assay at 24 h. (**h**) Effect of DAS and vitamin C on cell death using MTT (3-[4,5-dimethylthiazol-2-yl]-2,5-diphenyltetrazolium bromide) assay at 24 h. SVGA astrocytes were incubated with 100 mℳ ethanol after CYP2E1 siRNA or scramble siRNA transfection or in the absence and presence of DAS, vitamin C, or vitamin E. ROS measurements, caspase-3 cleavage activity, TUNEL assay, MTT assay, and SiRNA transfection are described in Materials and Methods. The ROS was presented as the percentage of mean fluorescence intensity (%MFI), with 100% (1000 MFI) normalized for each control. MFI for controls at different time points did not vary significantly. The cell death was also normalized as 100% for control (without any treatments). The caspase-3 activity was presented in absolute units. The green color in TUNEL assay represents DNA fragmentation. *P*≤0.05 (*) values were calculated by comparison of ethanol treatment with respective controls as presented.Mean±SD was calculated from at least three experiments and significance was determined using one-way analysis of variance

As previously shown in U937 monocytic cells,^15^ we examined whether ethanol also induces ROS in SVGA astrocytes at 100 mℳ ethanol (near physiological concentration in binge drinkers) at 12–36 h. Single treatment of 100 mℳ ethanol induced ROS production by >20% at 24 and 36 h (Figure 1a). Further, to examine whether CYP2E1 is responsible for the generation of ROS, we knocked down CYP2E1 expression through transfection using 10 nℳ predesigned CYP2E1 siRNA and 10 nℳ scrambled siRNA as control. In all, 10 nℳ CYP2E1 siRNA effectively diminished CYP2E1 protein expression (Figure 1b, right panel), which significantly reduced ethanol-induced formation of ROS at 24 h (Figure 1b, left side). Although not significant, CYP2E1 siRNA alone slightly increased ROS level compared with scramble siRNA. These results suggested the role of CYP2E1 in ethanol-induced ROS production in SVGA astrocytes.

As caspase-3 cleavage is a marker of early apoptosis, we examined caspase-3 cleavage activity at 100 mℳ ethanol treatment for 24 h in SVGA astrocytes. The results showed that ethanol increased caspase-3 cleavage activity by more than twofold compared with control. In addition, knocking down CYP2E1 expression through CYP2E1 siRNA almost completely abolished ethanol-induced caspase-3 cleavage (Figure 1c). Furthermore, diallyl sulfide (DAS), a selective chemical inhibitor of CYP2E1, which is also a food additive and has protective effect on immune cells,^16^ abolished ethanol-induced apoptosis (Figure 1d). In addition, 100 *μ*ℳ vitamin C, as well as vitamin E, blocked the effect of ethanol on induction of caspase-3 cleavage activity (Figures 1e and f), suggesting that ethanol-induced apoptosis is mediated through ROS production. Vitamin C alone also showed decreased caspase-3 cleavage activity compared with control, and it appeared to be more effective than vitamin E. However, other anti-oxidants tested, N-acetyl cysteine (NAC) and butylated hydroxyltoluene (BHT), did not reduce ethanol-induced apoptosis, rather they further induced caspase-3 cleavage activity in combination with ethanol (Supplementary Figure S1). Therefore, we used vitamin C as an antioxidant in subsequent experiments.

To further confirm the effect of ethanol, as well as the role of CYP2E1 and oxidative stress on apoptosis, terminal deoxynucleotidyl transferase dUTP nick end labeling (TUNEL) assay was performed in SVGA astrocytes. The results showed that 24 h ethanol treatment at 100 mℳ significantly increased formation of DNA fragments (Figure 1g). Although DAS alone showed some DNA fragmentation, both DAS and vitamin C effectively reduced ethanol-induced DNA fragmentation in SVGA astrocytes (Figure 1g). Finally, we tested whether DAS and antioxidant rescue ethanol-induced cell death using MTT assay. Ethanol showed a time- (12–48 h) and dose-dependent (100–200 mℳ) effect on the cell death of SVGA astrocytes (Supplementary Figures S2A and B). Further, 100 mℳ ethanol showed 27% cell death, which was rescued by DAS and vitamin C (Figure 1h). Similar to TUNEL assay, DAS alone caused ∼15% cell death compared with control. Although DAS has a protective effect,^16^ it is also known to cause toxicity at high concentration and when used for longer time.^17^ Therefore, we performed a subsequent experiment using CYP2E1 siRNA to assess the specificity of DAS. Similar to the increase in oxidative stress by CYP2E1 siRNA alone (Figure 1b), it also caused significant cell death (Supplementary Figure S2C), suggesting that a basal level of CYP2E1 is required for cell survival. In fact, a physiological role of CYP2E1 is documented in dopamine metabolism and nuclear factor-E2-related factor 2 induction in brain cells.^18, 19, 20^ However, as expected, CYP2E1 siRNA abolished ethanol-induced cell death (Supplementary Figure S2C). Overall, our results clearly suggested the role of CYP2E1 and ROS in ethanol-induced apoptosis and cell death in SVGA astrocytes.

### Upregulation of CYP2E1 expression by ethanol-mediated oxidative stress in SVGA astrocytes

**Figure 2:**
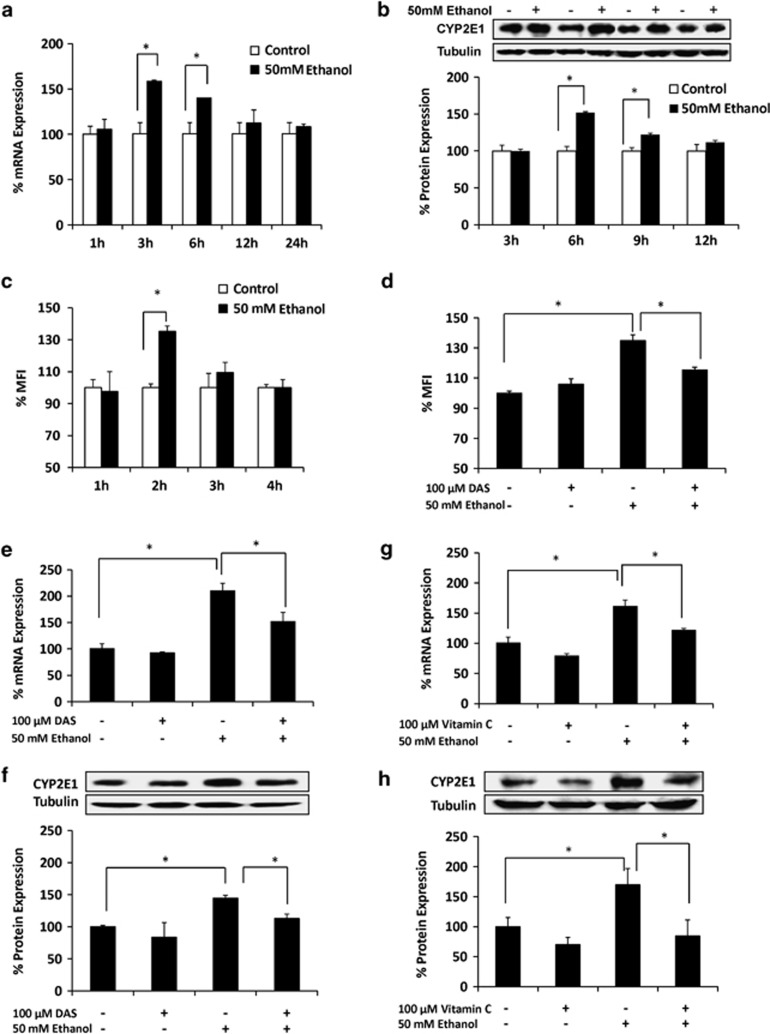
Upregulation of CYP2E1 expression by ethanol-mediated oxidative stress in SVGA astrocytes. Effect of 50 mℳ ethanol on CYP2E1 (**a**) mRNA and (**b**) Effect of 50 mM ethanol on ROS production at 1–4 h. (**c**) protein expressions at 1–24 h. (**d**) Effect of DAS in ethanol-mediated ROS production at 2 h. Effect of DAS on ethanol-induced CYP2E1 (**e**) mRNA and (**f**) protein expressions. Effect of vitamin C on ethanol-induced CYP2E1 (**g**) mRNA and (**h**) protein expressions. The mRNA and protein expression levels were evaluated at 3 and 6 h, respectively, and presented in percentage, with 100% expression normalized for the untreated cells at every time point. Expression of each gene was normalized using GAPDH (glyceraldehyde 3-phosphate dehydrogenase), while *β*-tubulin was used as an internal control for protein expression. The ROS was presented as the percentage of mean fluorescence intensity (%MFI), with 100% (1000 MFI) normalized for each control. MFI for controls at different time points did not vary significantly. *P*≤0.05 (*) values for each comparison are presented. Mean±SD was calculated from at least three experiments and significance was determined using one-way analysis of variance

The basal levels of mRNA expression of CYP enzymes were earlier detected in SVGA astrocytes.^21^ Compared with the two most abundant CYP enzymes, CYP2A6 (56%) and CYP1A1 (43%), CYP2E1 showed relatively low mRNA expression (3%). However, its relative level in SVGA astrocytes compared with other CYPs is similar to that in the liver.^22^ Further, as previously shown in U937 monocytic cells,^15^ we investigated whether ethanol induces CYP2E1 in SVGA astrocytes. Initial results showed that 50 mℳ ethanol is optimum to induce CYP2E1 for up to 24 h (data not shown). The ethanol concentration at ≥100 mℳ caused significant cell death in SVGA astrocytes (Supplementary Figures S2A and B). Therefore, we used 100 mℳ ethanol for oxidative stress, apoptosis, and cell death experiments at 24 h (Figure 1), while we used 50 mℳ ethanol for examining the induction of CYP2E1 in SVGA astrocytes (Figure 2). Kinetic profile of CYP2E1 expression showed that 50 mℳ ethanol resulted in significant upregulation of CYP2E1 mRNA at 3 h (>150%) and 6 h (<150%) compared with control (Figure 2a). Ethanol also showed 150% increased expression of CYP2E1 protein at 6 h, compared with control (Figure 2b). Both mRNA and protein expression levels of CYP2E1 decreased to the level of control at ≥12 h.

To examine whether CYP2E1 induction is associated with ethanol metabolism-mediated ROS production, we measured ROS production at early time points up to 4 h in the absence and presence of 50 mℳ ethanol in SVGA astrocytes (Figure 2c). The data showed that ROS production was increased at 2 h (>30%) by ethanol treatment. This result is consistent with the other observations, in which nicotine treatments also generated ROS at early time points (30 min–2 h) in SVGA astrocytes.^21^ To complement the finding in Figure 1a, we used 100 mℳ ethanol at 1 and 2 h, which showed higher increase in ROS (Supplementary Figure S3) than the ROS generated at 50 mℳ ethanol (Figures 2c and d). As expected, CYP2E1 selective inhibitor, DAS, significantly decreased ethanol-induced oxidative stress at 2 h (Figure 2d), suggesting the role of CYP2E1 in the production of ROS by ethanol metabolism. Furthermore, to determine whether CYP2E1-mediated ethanol metabolism and subsequent ROS production are responsible for CYP2E1 induction, SVGA astrocytes were pretreated with 100 *μ*ℳ DAS and vitamin C followed by ethanol treatment for 6 h. DAS significantly reduced ethanol-mediated CYP2E1 induction at both mRNA and protein levels (Figures 2e and f). Similarly, 100 mℳ vitamin C also abolished ethanol-mediated induction of CYP2E1 mRNA as well as protein (Figures 2g and h). DAS and vitamin C alone did not alter CYP2E1 expression significantly. In order to confirm that CYP2E1 is the primary enzyme responsible for ethanol metabolism in SVGA astrocytes, we measured ADH mRNA in astrocytes. However, the level of ADH in SVGA astrocytes was undetectable. These results suggested that ethanol-induced CYP2E1 expression is mediated through CYP2E1-mediated ethanol metabolism and subsequent production of ROS.

### Regulation of CYP2E1 expression by ethanol through PKC/JNK/SP1 pathway in SVGA astrocytes

**Figure 3:**
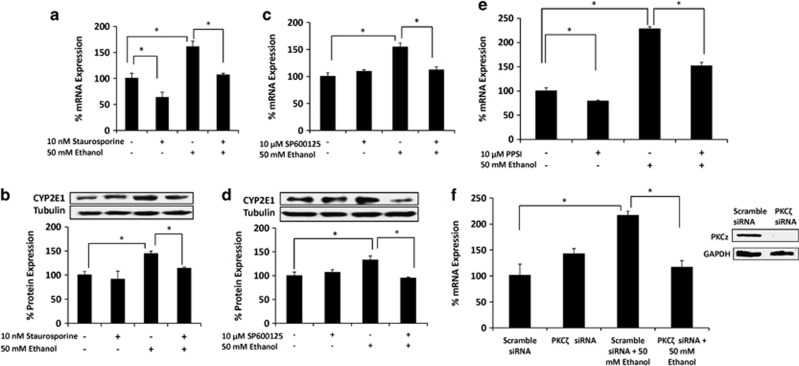
Role of PKC*ζ*/JNK pathway on ethanol-mediated CYP2E1 induction in SVGA astrocytes. Effect of staurosporine (PKC inhibitor) on ethanol-induced CYP2E1 (**a**) mRNA and (**b**) protein expressions. Effect of SP600125 (JNK inhibitor) on ethanol-induced CYP2E1 (**c**) mRNA and (**d**) protein expressions. Effect of (**e**) PPSI (PKC*ζ* inhibitor), and (**f**) PKC*ζ* small interfering RNA (siRNA) on CYP2E1 mRNA expression. mRNA and protein expressions were evaluated at 3 and 6 h, respectively, in the presence of 100 mℳ ethanol (−/+ inhibitors or siRNA). The inhibitors' treatment and siRNA transfection are described in Materials and Methods. The mRNA and protein expression levels are presented in percentage, with 100% expression normalized for the untreated cells. Expression of each gene was normalized using glyceraldehyde 3-phosphate dehydrogenase (GAPDH), while *β*-tubulin or GAPDH was used as an internal control for protein expression. *P*≤0.05 (*) values for each comparison are presented. Mean±SD was calculated from at least three experiments and significance was determined using one-way analysis of variance

To determine the underlying mechanism responsible for ethanol-mediated CYP2E1 induction, SVGA astrocytes were pretreated with staurosporine, an inhibitor of protein kinase C (PKC), as well as inhibitors of c-Jun N-terminal kinase (JNK) inhibitor (SP600125) and mitogen-activated protein kinase kinase (MEK) inhibitor (U0126). Staurosporine abrogated ethanol-mediated induction of CYP2E1 mRNA and protein (Figures 3a and b). In addition, while JNK inhibitor abolished ethanol-mediated CYP2E1 induction (Figures 3c and d), the MEK inhibitor showed no effect (Supplementary Figure S4A). Furthermore, as PKC*ζ* is the major subtype of PKC family that mediates JNK activation,^23^ we tested whether selective inhibitor of PKC*ζ* (PKC*ζ* pseudo-substrate inhibitor (PPSI)), as well as PKC*ζ* siRNA, abrogates ethanol-mediated CYP2E1 expression in SVGA astrocytes. As expected, 10 *μ*ℳ PPSI significantly reduced ethanol-induced CYP2E1 mRNA expression (Figure 3e) and 10 nℳ PKC*ζ* siRNA completely blocked ethanol-induced CYP2E1 mRNA expression (Figure 3f). Overall, these results suggest that the expression of CYP2E1 is regulated by the activation of the PKC/JNK pathway.

**Figure 4:**
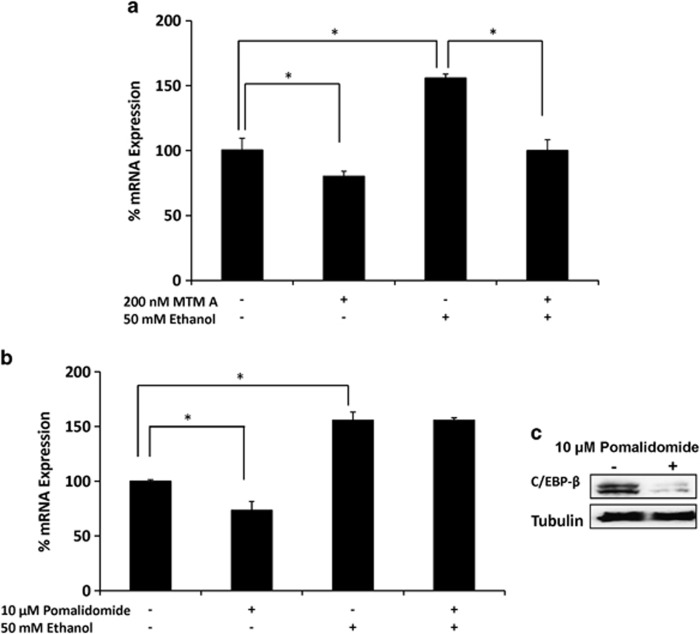
Role of SP1 transcription factor on ethanol-mediated CYP2E1 induction in SVGA astrocytes. Effect of (**a**) mithrimycin A (SP1 inhibitor) and (**b**) pomalidomide (C/EBP *β* inhibitor) on ethanol-induced CYP2E1 mRNA expression. (**c**) Effect of pomalidomide on C/EBP-*β* protein expression. The mRNA expression level was evaluated at 3 h in the presence of 50 mℳ ethanol (−/+ mithrimycin A or pomalidomide). The results are provided in percentage, in which 100% expression was normalized for the control. *P*≤0.05 (*) values for each comparison are presented. Mean±SD was calculated from at least three experiments and significance was determined using one-way analysis of variance

To further examine the transcription factor that is involved in ethanol-mediated CYP2E1 induction, 10 *μ*M pomalidomide, a selective inhibitor of CCAAT/enhancer-binding protein-*β* (C/EBP-*β*), and 200 nM mithrimycin A, a selective inhibitor of specificity protein 1 (SP1), were used in SVGA astrocytes, followed by treatment with 50 mM ethanol. Although mithrimycin A alone slightly downregulated CYP2E1 mRNA expression, it completely abolished ethanol-mediated induction of CYP2E1 (Figure 4a). By contrast, although pomalidomide alone also reduced CYP2E1 expression, it did not alter ethanol-mediated induction of CYP2E1 (Figure 4b), in spite of the fact that pomalidomide reduced C/EBP-*β* protein expression (Figure 4c). Thus, our results suggest that SP1 is responsible for the regulation of CYP2E1.

### Role of CYP2E1 in oxidative stress-mediated cell death by ethanol in U937 monocytes

**Figure 5:**
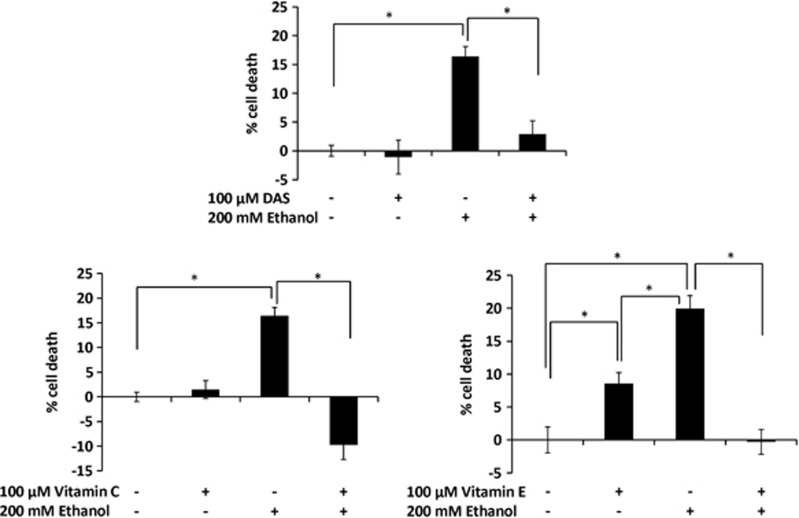
Role of CYP2E1 in oxidative stress-mediated cell death by ethanol in U937 monocytes. Bar graphs represent percentage of cell death using MTT (3-[4,5-dimethylthiazol-2-yl]-2,5-diphenyltetrazolium bromide) assay. 200 mℳ ethanol treatment was performed in the absence and presence of DAS, vitamin C, or vitamin E, followed by measurement of cell death (MTT assay) after 48 h. MTT assays are described in Materials and Methods. *P*≤0.05 (*) values for each comparison are presented. Mean±SD was calculated from at least three experiments and significance was determined using one-way analysis of variance

As shown in SVGA astrocytes (Figures 1c–g), we examined the role of CYP2E1 and vitamin C on apoptosis in U937 monocytes using annexin V assay under different conditions with respect to treatment times and ethanol concentrations. Ethanol showed a minor increase in apoptosis, which to some extent, was rescued by DAS, vitamin C, and vitamin E (Supplementary Figure S5). However, the changes in these results were not conclusive. Furthermore, we performed cell death assay using 200 mℳ ethanol at 48 h (optimal conditions), which showed >15% cell death (Figure 5). As expected 100 *μ*ℳ DAS as well as 100 *μ*ℳ antioxidants (vitamins C and E) both rescued cell death induced by 100 mℳ ethanol (Figure 5). Unlike SVGA astrocytes (Figure 1), DAS did not cause significant cell death in U937 monocytes (Figure 5). However, similar to SVGA astrocytes, vitamin C was relatively more effective than vitamin E in U937 monocytes.

### Regulation of CYP2E1 expression by ethanol through oxidative stress-mediated PKC/JNK/SP1 pathway in U937 monocytes

**Figure 6:**
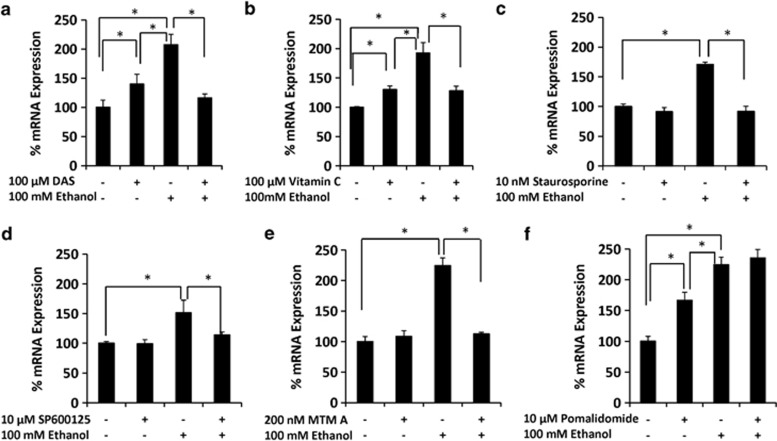
Role of PKC/JNK/SP1 pathway on ethanol-mediated CYP2E1 induction in U937 monocytes. Effect of (**a**) DAS, (**b**) vitamin C, (**c**)staurosporine, (**d**) SP60012, (**e**) mithrimycin A, and (**f**) pomalidomide on ethanol-induced CYP2E1 mRNA expression. These data were evaluated at 12 h in the presence of 100 mℳ ethanol. The results are provided in percentage, in which 100% expression was normalized for the control. *P*≤0.05 (*) values for each comparison are presented. Mean±SD was calculated from at least three experiments and significance was determined using one-way analysis of variance

**Figure 7:**
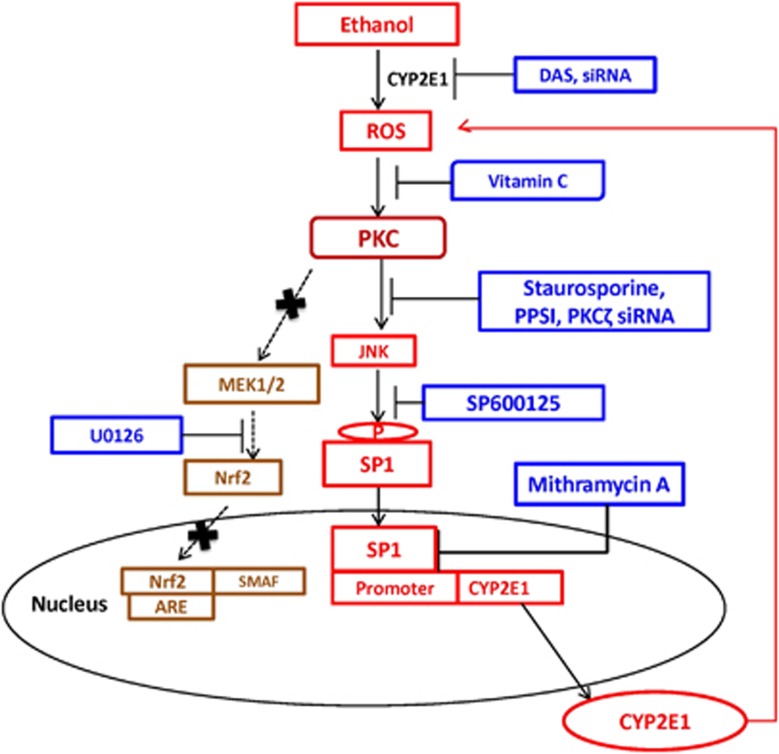
Schematic representation of CYP2E1-mediated ROS production by ethanol, followed by activation of PKC*ζ*/JNK/SP1 pathway for the expression of CYP2E1 (red). Inhibitors that inhibit specific pathway of CYP2E1 expression are presented in blue. ARE, anti-oxidant response element; Nrf2, nuclear factor-E2-related factor 2; SMAF, small molecule activation factor

As in SVGA astrocytes, we investigated the mechanism by which CYP2E1 is regulated by ethanol in U937 monocytes. The results showed that DAS and vitamin C inhibited ethanol-induced CYP2E1 mRNA expression (Figures 6a and b). Treatment of U937 cells with either DAS or vitamin C also slightly increased CYP2E1 expression. Similarly, PKC inhibitor (staurosporine), JNK inhibitor (SP600125), and SP1 inhibitor (Mithramycin A), completely abolished ethanol-induced CYP2E1 mRNA expression (Figures 7c–e). These inhibitors did not show any effect on the basal levels of CYP2E1 expression. Similar to SVGA astrocytes, MEK inhibitor (U0126) (Supplementary Figure S4B) and C/EBP-*β* inhibitor (pomalidomide) (Figure 4b), these inhibitors did not alter induction of CYP2E1 mRNA expression by ethanol in U937 monocytes (Figure 6f). Thus, the expression of CYP2E1 is also regulated by oxidative stress-mediated activation of PKC/JNK/SP1 pathway in U937 monocytes.

## Discussion

Several previously reported *in vitro* and *in vivo* studies have shown that both acute and chronic alcohol consumptions increase CYP2E1 expression, leading to liver toxicity.^2, 8, 24, 25, 26, 27, 28^ Although ethanol-mediated CYP2E1 induction, as well as CYP2E1-mediated oxidative damage through ethanol metabolism, is well established in the liver,^2, 8, 29^ the mechanistic pathways in ethanol-associated CYP2E1 induction in hepatic as well as extra-hepatic cells remain unclear. This is the first report to provide strong evidence of the involvement of the PKC/JNK/SP1 pathway in ethanol-mediated regulation of CYP2E1 in astrocytes and monocytes (Figure 7). This is also the first report showing the role of CYP2E1 in oxidative stress-mediated apoptotic cell death in these extra-hepatic cells.

CYP2E1 has been found to be the major alcohol-metabolizing enzyme in the brain, and it is associated with oxidative damage in the brain.^10, 30^ CYP2E1 has also been shown to have a crucial role in ethanol-mediated lipid peroxidation in the brain, leading to increased permeability of BBB and dysfunction of mitochondria.^10, 11^ Consistent with these observations, our previous study has shown that ethanol upregulates CYP2E1 in the U937 cell line and its expression is associated with increased oxidative stress.^15^ As the level of ADH is undetectable in U937 cells, CYP2E1 has been suggested to be the major enzyme responsible in ethanol-mediated oxidative stress in monocytes.^15^ Similarly, in the present study, we demonstrated the upregulation of CYP2E1 by ethanol in SVGA astrocytes. Furthermore, we showed that CYP2E1 is responsible for ethanol-mediated ROS production and apoptotic cell death in SVGA astrocytes as well as in U937 monocytes.

Our observation that acute ethanol treatment induces CYP2E1 expression by approximately 1.5-fold in SVGA astrocytes is significant and consistent with our earlier observation in U937 cells,^15^ as well as with observations from other studies.^31, 32^ However, in primary monocytes of chronic alcohol users, CYP2E1 mRNA expression showed ∼10-fold induction (unpublished observations) compared with healthy individuals, which is consistent with hepatic CYP2E1 induction in chronic alcohol users.^27, 33^ Persistent induction of CYP2E1 by alcohol consumption in chronic users is known to enhance the formation of ROS, which inhibits acetaldehyde dehydrogenase resulting in accumulation of acetaldehyde.^4^ In addition to ROS, which is known to damage DNA and protein, acetaldehyde is associated with decreased DNA repair, impaired hepatic utilization of oxygen, and an increase of glutathione depletion.^4^ Accumulation of acetaldehyde is known to have a key role in ethanol-induced brain damage.^34^ As ADH is not involved in alcohol metabolism in the brain,^10^ CYP2E1 appears to have the dominant role in ethanol-mediated brain damage. Our results from astrocytes and monocytes, which are the major cell types required for brain function, lend further support to this hypothesis.

Increased oxidative stress by CYP2E1 induction is known to be a major consequence of ethanol-mediated liver toxicity.^2^ A single dose of ethanol is found to induce superoxide dismutase, catalase, and glutathione *S*-transferase as a result of production of ROS, which protect against oxidative stress.^29^ However, chronic alcohol exposure leads to decreased expressions of superoxide dismutase and catalase,^35, 36^ while alcohol-mediated CYP2E1 induction and subsequent alcohol metabolism lead to further increase in production of ROS and acetaldehyde, especially in mitochondria.^37^ Our observations suggested that ethanol induces ROS production (through CYP2E1-mediated ethanol metabolism), leading to the induction of CYP2E1, which further produces ROS, causing cell apoptosis and death. Our results on the effect of vitamins C and E are consistent with the observations that the use of antioxidant supplements, such as vitamins C and E, provides therapeutic effects by attenuating oxidative stress-mediated alcohol-induced liver diseases.^38^ In our study, vitamins C and E both abrogated ethanol-mediated apoptosis and cell death in both astrocytes and monocytes. Thus, our study also supports the use of antioxidants, especially vitamin C, in preventing alcohol-mediated cell toxicity.

Alcohol-mediated oxidative stress has been shown to induce antioxidant enzymes through the PKC signaling pathway to negate the effects of oxidative stress.^39, 40^ However, consistent use of alcohol is also known to cause alcohol-induced toxicity and liver damage through the PKC pathway.^41^ Our results are consistent with the observation that ethanol-mediated oxidative stress induces CYP2E1 through the PKC pathway, which further metabolizes ethanol and produces ROS (Figure 7). The activation of PKC by increased oxidative stress leads to phosphorylation of downstream proteins and induction of downstream signaling cascades.^39, 40, 41^ Previous studies have shown that ethanol can induce multiple signaling cascades and transcription factors, such as mitogen-associated protein kinase and nuclear factor kappa-light-chain-enhancer of activated B cells (NF-*κ*B), which have important roles in cytokine release and the induction of inflammation.^42^ Other studies have shown that lipopolysaccharide-mediated CYP2E1 induction in astrocytes is associated with activation of MEK3 and C/EBP-*β*,^43^ while in hepatocytes both SP1 and NF-*κ*B are involved in regulation of CYP2E1.^44^ However, our study clearly demonstrates the role of the PKC/JNK/SP1 pathway in ethanol-mediated regulation of CYP2E1 expression (Figure 7).

Staurosporine is known to bind PKC, leading to inhibition of phosphorylation of MEK and JNK proteins.^41, 45^ Our results using staurosporine and SP600125 (JNK inhibitor) clearly showed that phosphorylation of JNK, but not MEK, regulates ethanol-mediated CYP2E1 induction in U937 monocytes and SVGA astrocytes. Consistent with the previous observation,^23^ our finding also suggest that PKC*ζ* is the major subtype of PKC family that mediates JNK activation. With regard to the involvement of transcription factors in CYP2E1 induction, c-Jun has been previously reported to bind to C/EBP-*β* and act as a transcriptional activator.^46^ C/EBP-*β* is also known to be involved in both interleukin (IL)-4-mediated CYP2E1 regulation and cell apoptosis.^47, 48^ Further, SP1 transactivation, which is also known to interact with c-Jun, has been shown to bind to the promoter of CYP2E1,^44^ as well as being involved in ethanol-mediated induction of heat-shock protein 70.^49^ Consistent with these observations, our results clearly show that SP1, but not C/EBP-*β*, is involved in PKC/JNK-mediated regulation of CYP2E1 expression in astrocytes and monocytes. Our finding of the association of JNK with ethanol-mediated CYP2E1 induction has implications in targeting the JNK/SP1 pathway for novel therapeutic intervention for the treatment of neurotoxicity in alcohol users.

In addition to CYP2E1 (our study), pro-inflamatory cytokines, such as IL-1*β* and tumor necrosis factor-*α*, are also induced by alcohol.^50, 51^ Alcohol-mediated upregulation of pro-inflamatory cytokines occurs through the MAP kinase pathway (ERK1/2, p-38, and JNK), which triggers the downstream activation of oxidant-sensitive transcription factors NF-*κ*B and AP-1.^50^ These pathways are associated with an increased apoptosis in ethanol-fed rats (cerebral cortex) and in ethanol-treated astrocytes, suggesting that chronic ethanol treatment stimulates glial cells by upregulating pro-inflammatory cytokines through the signaling pathways involved in cell death.^50, 51^ Previous study has shown that anti-inflamatory cytokine IL-4 can induce CYP2E1 in hepatic cells through PKC pathway.^47^ Taken together, it can be suggested that there is a crosstalk between CYP2E1 and cytokines in alcohol-mediated neuronal toxicity. These findings have important implications for inflammation in both the periphery and the CNS in the case of simultaneous exposure to alcohol and infection with bacterial or viral pathogens. Therefore, further dissection of the signaling pathways that are responsible for CYP2E1 induction and cytokine release is imperative to further our understanding of ethanol-mediated toxicity in monocytes and astrocytes.

The present study suggests that elevated oxidative stress by ethanol is not only the consequence, but also the mediator, of CYP2E1 induction in astrocytes and monocytes. Furthermore, an increased CYP2E1 expression and resultant oxidative stress cause apoptotic cell death in these cells, suggesting that CYP2E1, in addition to oxidative stress, is one of the key players to target alcohol-mediated brain toxicity. Attenuation of CYP2E1-mediated apoptosis-dependent cell death of monocytes, lymphocytes, and neurons is expected to help attenuate alcohol-mediated immune suppression and neurotoxicity. DAS, a selective inhibitor of CYP2E1, is a food additive, and has been shown to be protective to immune cells,^16^ could be a potential target for alcohol-induced immune suppression and neurotoxicity. However, as DAS could also be toxic,^17^ novel chemical derivatives with relatively lower toxicity than DAS can be synthesized to use them as a therapeutic.

## Materials and Methods

### Materials

The U937 monocytic cell line was obtained from ATCC (Manassas, VA, USA). The SVGA astrocyte cell line was generously provided by Dr. Avindra Nath, NIH/NIDA. DAS, vitamin C, vitamin E, staurosporine, U0126, SP600125, pomalidomide, and protease inhibitor cocktail, NAC and BHT were bought from Sigma-Aldrich, St. Louse, MO, USA. Roswell Park Memorial Institute (RPMI) 1640 and Dulbecco's Modified Eagle Medium (DMEM) media were purchased from Mediatech Inc., Manassas, VA, USA. Qiagen RNeasy kit was obtained from Qiagen, Valencia, CA, USA. Gene expression kit and primer probes were obtained from Applied Biosystems (Carlsbad, CA, USA). MTT proliferation assay and mithramycin A were from R&D systems, Inc. (Minneapolis, MN, USA). TUNEL apoptosis and Annexin V/PE apoptosis kits were from Genscript Inc. (Piscataway, NJ, USA) and BD Biosciences (San Jose, CA, USA), respectively. BCA protein assay kit was purchased from Thermo Scientific (Rockford, IL, USA). Dichlorofluoroscein diacetate (DCFDA) was purchased from Invitrogen (Grand Island, NY, USA). Radioimmunoprecipitation assay buffer and protease inhibitor cocktail were bought from Boston Bioproducts (Ashland, MA, USA). Primary and secondary antibodies were purchased from Santa Cruz Biotechnology Inc. (Santa Cruz, CA, USA). Scramble, predesigned CYP2E1, and PKC*ζ* siRNA, as well as lipofectamine, were purchased from Life Technologies (Grand Island, NY, USA). PPSI was obtained from Santa Cruz Biotechnology, Inc. Caspase-3 colorimetric assay kit was from Clontech Laboratories, Inc. (Mountain View, CA, USA).

### Cell culture and treatments

U937 cells were grown in RPMI 1640 media with 1% gentamicin at 37 °C in a humidified incubator containing 5% CO_2_. SVGA cells were grown in DMEM, containing 1% gentamicin. Ethanol treatment of monocytes was performed as previously described,^52^ and samples treated at an ethanol concentration of 100 mℳ for 12 h were selected for inhibitor studies. Ethanol treatment of astrocytes was performed 12 h after seeding cells in six-well plates. Desiccator-like containers containing 150 ml of 100 mℳ ethanol were pre-incubated for 1 h for ethanol saturation. Then, 50 mℳ ethanol was added to each well, and the plates within the ethanol-saturated containers were incubated in the incubator. Both ethanol concentrations (10–50 mℳ) and time course (1–24 h) were optimized for ethanol-mediated CYP2E1 induction. A dose of 50 mℳ ethanol at 3 h was found to be optimal for further experiments in the case of astrocytes. Treatments with vitamin C, vitamin E, NAC, BHT, staurosporine, U0126, SP600125, pomalidomide, and mithrimycin were initiated 1 h before ethanol treatment. However, cells treated with DAS were pre-treated for 15 min, before ethanol treatment according to the previous protocol.^53^ We used controls for each time point with or without these agents.

### ROS measurement by flow cytometry

The production of ROS was measured by flow cytometry using DCFDA as previously described.^24^ Briefly, the astrocytes were treated with alcohol, either with or without inhibitors, using serum-free medium at different times in a six-well plate followed by addition of 10 *μ*ℳ DCFDA. Cells were then harvested and dissolved in 1 ml PBS to measure the DCF emission at 525±20 nm by flow cytometry and mean fluorescence intensity was measured and analyzed.

### Apoptotic assay

Caspase 3 cleavage activity was measured according to the manufacturer's protocol. Briefly, 2 × 10^6^ cells were collected and resuspended in 50 *μ*l cell lysate buffer for 10 min on ice and then centrifuged at maximum speed for 10 min at 4 °C. Further, 50 *μ*l reaction reagent, including 1% DTT, was then added to each supernatant and mixed properly. After adding 5 *μ*l caspase 3 substrate individually, samples were kept in the water bath at 37 °C for 1 h. Samples were then measured using microplate reader at 405 nm.

A TUNEL apoptosis test was applied in SVGA astrocytes, which were adherent, to measure cellular apoptosis induced by treatments. Cells in each well were cultured on a cover slip. After termination of treatments, cells were fixed in fresh 4% formaldehyde for 30 min and then incubated with 70% ethanol for 30 min to increase membrane permeability. After incubation with permeabilization solution on ice for 2 min, cells were labeled through incubation with labeling solution (containing 2% FITC-12-dUTP) for 1 h in the dark. After washing, cells were mounted on a slide and fluorescence was detected using a confocal microscope with an emission wavelength of 515 nm.

Annexin V detection was performed in U937 monocytes to measure the effect of ethanol and inhibitors on apoptosis and cell death. Briefly, media was removed and cells from each well were suspended in binding solution at a final concentration of 1 × 10^6^ cells per ml, 100 *μ*l of which was transferred into a 5-ml tube. In all, 5 *μ*l PE, as indicator of early apoptosis, and 5 *μ*l of 7-AAD were added to the 100 *μ*l cell solution, followed by 15 min incubation at room temperature in dark. After incubation, 400 *μ*l binding solution was added to each tube and fluorescence was detected using flow cytometer (BD Biosciences). Mean fluorescence intensity was measured and analyzed.

### MTT assay

Cell viability test was performed on six-well plates, using MTT assay. Media was removed from each well to terminate cell treatments. After washing twice with PBS, cells from each well were incubated for 4 h with a mixture of 200 *μ*l 0.5 mg/ml MTT solution in PBS and 300 *μ*l fresh media. This media was then replaced by 500 *μ*l of a mixture of 400 *μ*l DMSO and 100 *μ*l Sorenson's glycine buffer. Absorbance was obtained at an emission wavelength of 570 nm in micro plate reader. The total number of cells remaining alive in each well was calculated using a standard curve.

### RNA extraction and qRT–PCR

Total RNA was extracted using Qiagen RNeasy kit based on the manufacturer's protocols. For each reaction, RNA (100 ng) from the samples was reverse-transcribed into cDNA using High-capacity cDNA Reverse Transcription Kit. qRT-PCR was performed using cDNA generated from the reverse transcription of RNA according to the manufacturer's instructions (TaqMan Gene Expression Kit, Applied Biosystems). PCR reactions were performed on the iCycler iQ system. (Bio-Rad Laboratories, Hercules, CA, USA). Relative gene expression was calculated using GAPDH as an endogenous control.

### Western blotting

Total cell lysates were prepared using radioimmunoprecipitation assay buffer, containing 1 × protease inhibitor cocktail. The protein concentrations were measured using BCA protein assay kit. Western blotting was performed essentially as described.^24^ Briefly, 20 *μ*g of total proteins were run on SDS-PAGE and then transferred to polyvinylidene fluoride membranes. Transferred blots were blocked in 5% nonfat dry milk followed by overnight incubation with primary antibody (1 : 1000) and 2-h incubation with an appropriate secondary antibody (1 : 1500). Proteins were detected by LuminataTM crescendo western HRP substrate (Millipore corporation, Billerica, MA, USA), using the Alpha Innotech FluorChem HD2 gel documentation system (Alpha Innotech, San Leandro, CA, USA). The densitometry data were analyzed using AlphaEase FC StandAlone software (version 6.0.0.14; Alpha Innotech). *β*-Tubulin or GAPDH served as internal loading control to normalize the expression of proteins.

### siRNA transfection

SVGA astrocytes were transfected with predesigned human CYP2E1 siRNA or scramble siRNA (10 nℳ) in six-well plates for 24 h with lipofectamine transfection reagent in serum-free and antibiotic-free media. Transfection media were then discarded, and SVGA astrocytes were incubated overnight with complete media (containing 10% FBS and 1% gentamicin), followed by 24 h ethanol treatment at 100 mℳ.

### Statistical analysis

Statistical analysis for qRT-PCR, western blotting, ROS measurement, caspase-3 cleavage activity, and MTT assay was performed to determine mean±SD. One-way ANOVA was applied to determine *P* values. A *P* value of ≤0.05 was considered significant.

## Abbreviations

CYP: cytochrome P450
ROS: reactive oxygen species
ADH: alcohol dehydrogenase
DAS: diallyl sulfide
TUNEL: terminal deoxynucleotidyl transferase dUTP nick end labeling
PKC: protein kinase C
JNK: c-Jun N-terminal kinase
MEK: mitogen-activated protein kinase kinase
SP1: specificity protein 1
C/EBP-*β*: CCAAT/enhancer-binding protein-*β*
