# Supplementary material for: Regulation of cytochrome P450 2e1 expression by ethanol: role of oxidative stress-mediated pkc/jnk/sp1 pathway
Source: Cell Death Dis. 2013 Mar 21;4(3):e554–. doi: 10.1038/cddis.2013.78 (PMC3615729; doi:10.1038/cddis.2013.78)
Supplement: Supplementary Information [file cddis201378x1.ppt]

## Slide 1
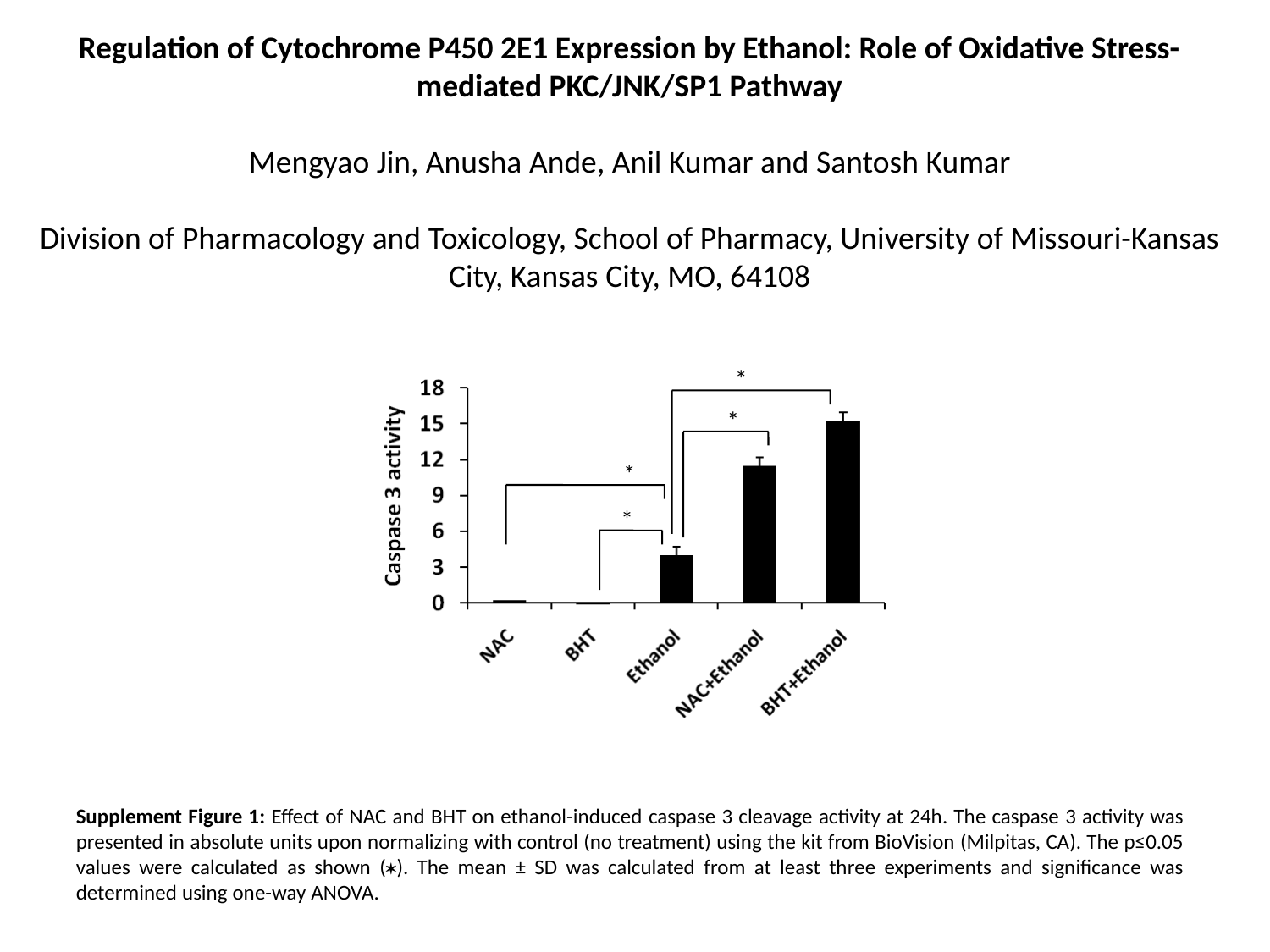

Regulation of Cytochrome P450 2E1 Expression by Ethanol: Role of Oxidative Stress-mediated PKC/JNK/SP1 Pathway
Mengyao Jin, Anusha Ande, Anil Kumar and Santosh Kumar
Division of Pharmacology and Toxicology, School of Pharmacy, University of Missouri-Kansas City, Kansas City, MO, 64108
*
*
*
*
Supplement Figure 1: Effect of NAC and BHT on ethanol-induced caspase 3 cleavage activity at 24h. The caspase 3 activity was presented in absolute units upon normalizing with control (no treatment) using the kit from BioVision (Milpitas, CA). The p≤0.05 values were calculated as shown (). The mean ± SD was calculated from at least three experiments and significance was determined using one-way ANOVA.

## Slide 2
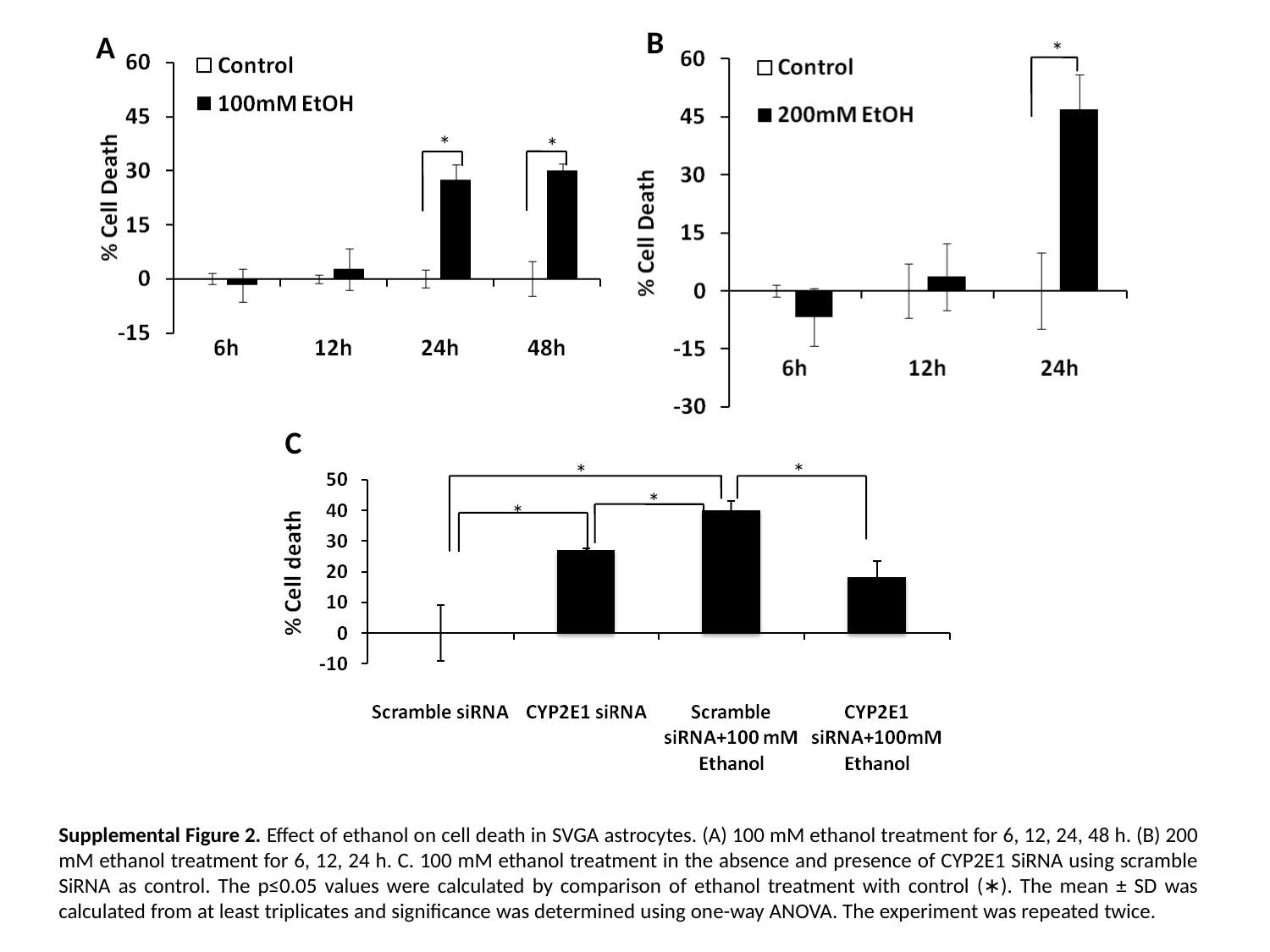

B
A
*
*
*
C
*
*
*
*
Supplemental Figure 2. Effect of ethanol on cell death in SVGA astrocytes. (A) 100 mM ethanol treatment for 6, 12, 24, 48 h. (B) 200 mM ethanol treatment for 6, 12, 24 h. C. 100 mM ethanol treatment in the absence and presence of CYP2E1 SiRNA using scramble SiRNA as control. The p≤0.05 values were calculated by comparison of ethanol treatment with control (∗). The mean ± SD was calculated from at least triplicates and significance was determined using one-way ANOVA. The experiment was repeated twice.

## Slide 3
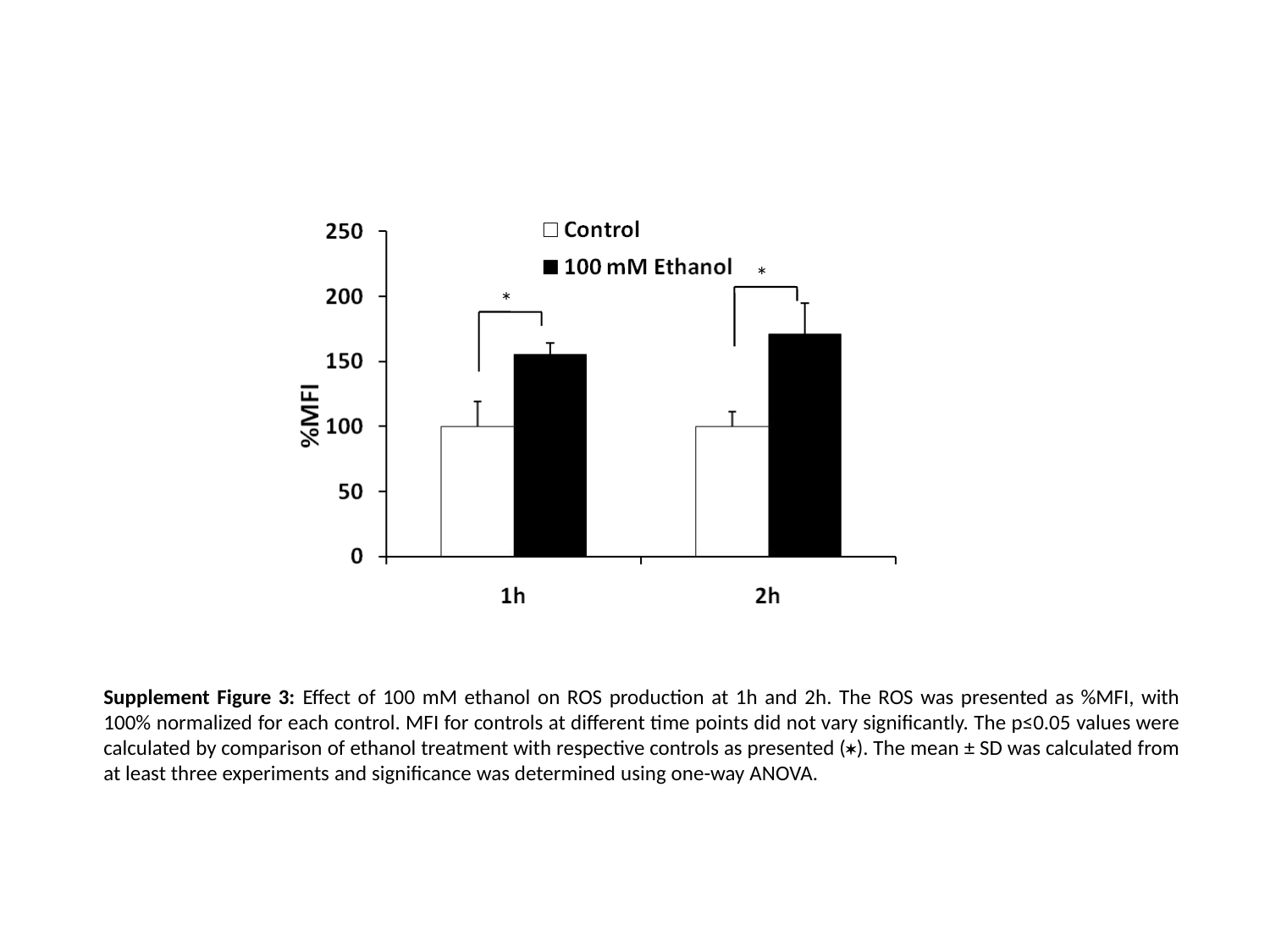

*
*
Supplement Figure 3: Effect of 100 mM ethanol on ROS production at 1h and 2h. The ROS was presented as %MFI, with 100% normalized for each control. MFI for controls at different time points did not vary significantly. The p≤0.05 values were calculated by comparison of ethanol treatment with respective controls as presented (). The mean ± SD was calculated from at least three experiments and significance was determined using one-way ANOVA.

## Slide 4
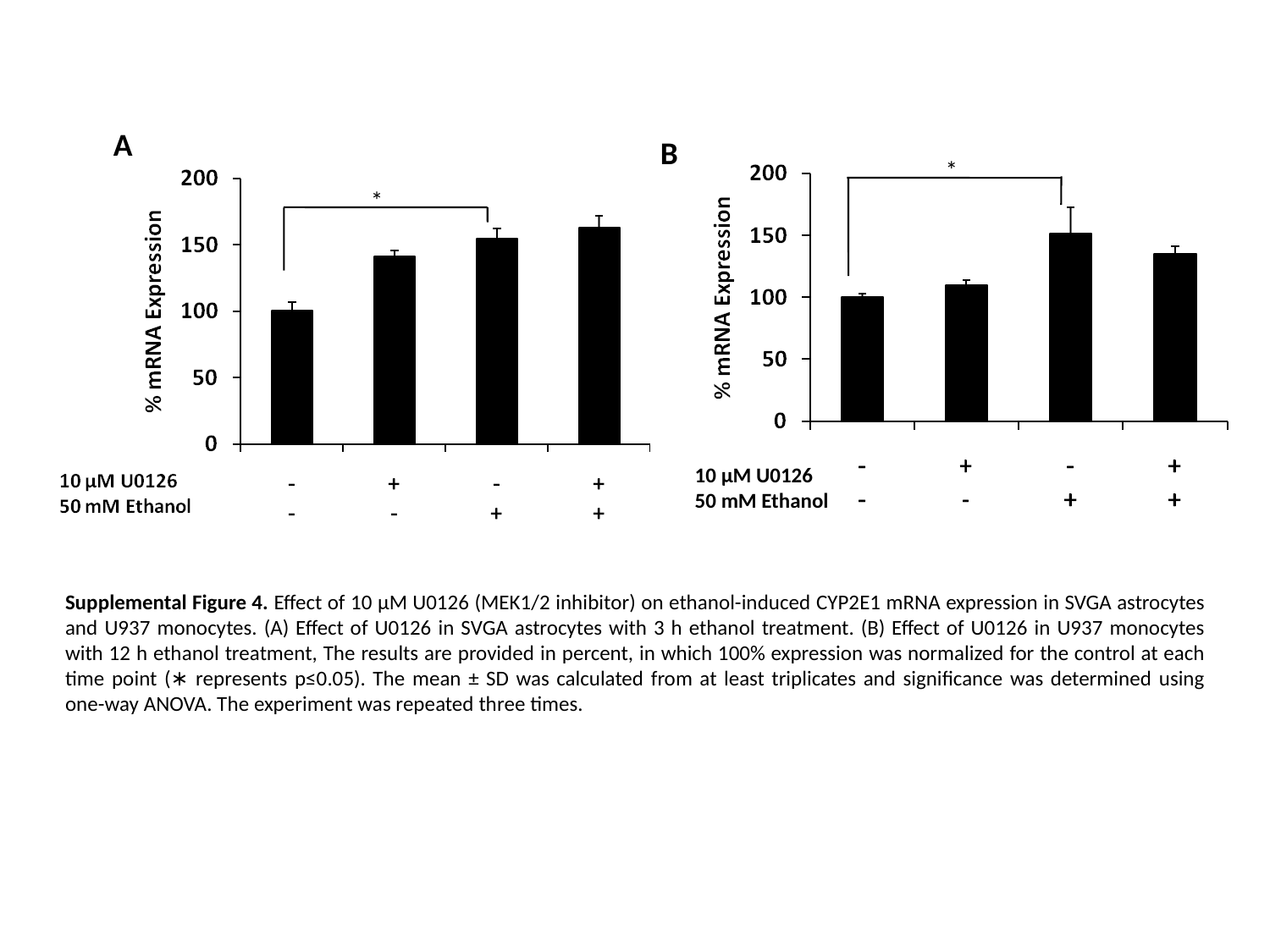

A
B
*
*
10 μM U0126
50 mM Ethanol
Supplemental Figure 4. Effect of 10 μM U0126 (MEK1/2 inhibitor) on ethanol-induced CYP2E1 mRNA expression in SVGA astrocytes and U937 monocytes. (A) Effect of U0126 in SVGA astrocytes with 3 h ethanol treatment. (B) Effect of U0126 in U937 monocytes with 12 h ethanol treatment, The results are provided in percent, in which 100% expression was normalized for the control at each time point (∗ represents p≤0.05). The mean ± SD was calculated from at least triplicates and significance was determined using one-way ANOVA. The experiment was repeated three times.

## Slide 5
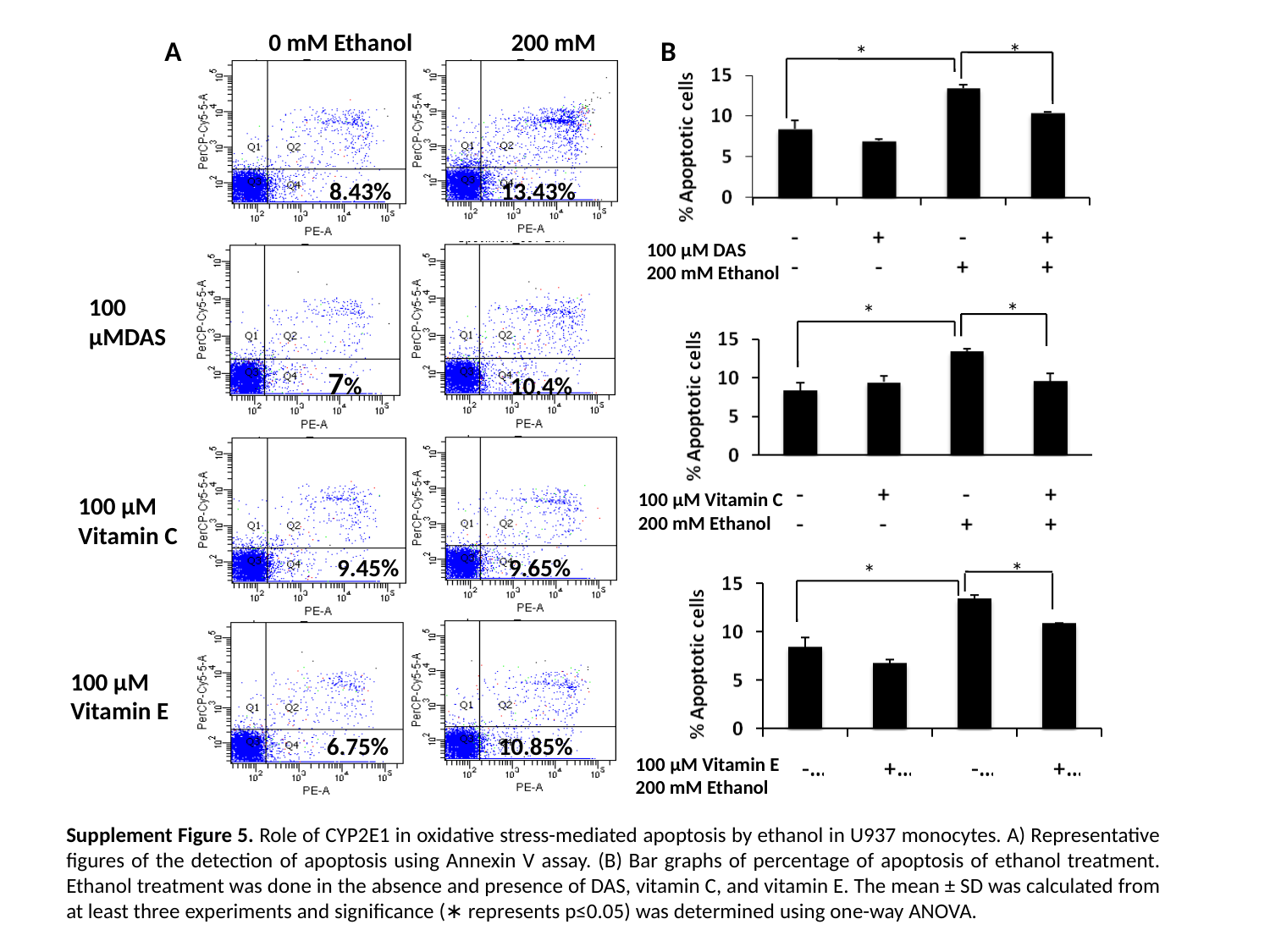

0 mM Ethanol 200 mM Ethanol
A
B
*
*
8.43% 13.43%
100 μM DAS
200 mM Ethanol
100 μMDAS
*
*
7% 10.4%
100 μM Vitamin C
200 mM Ethanol
100 μM
Vitamin C
9.45% 9.65%
*
*
100 μM
Vitamin E
6.75% 10.85%
100 μM Vitamin E
200 mM Ethanol
Supplement Figure 5. Role of CYP2E1 in oxidative stress-mediated apoptosis by ethanol in U937 monocytes. A) Representative figures of the detection of apoptosis using Annexin V assay. (B) Bar graphs of percentage of apoptosis of ethanol treatment. Ethanol treatment was done in the absence and presence of DAS, vitamin C, and vitamin E. The mean ± SD was calculated from at least three experiments and significance (∗ represents p≤0.05) was determined using one-way ANOVA.
